# Study on the shielding effect of low resistance body when using transient electromagnetic to detect urban underground space

**DOI:** 10.1371/journal.pone.0289184

**Published:** 2023-11-30

**Authors:** Yin An, Wang Yong, Chenyang Liu, Zhengcai Li, Xiaopei Zhang, Lizhi Du

**Affiliations:** College of Construction Engineering, Jilin University, Changchun, China; Southwest Jiaotong University, CHINA

## Abstract

Transient electromagnetic Method (TEM) is an efficient geophysical detection technology suitable for detection of urban near-surface space. However, its detection results are well affected by the low resistance anomaly, which interferes with the interpretation of the inversion results. This article used finite element method to simulate the entire process of urban underground pipeline under TEM detection. The causes of interference and the degree of interference under different working conditions were analyzed. The results demonstrate that low resistance anomaly in magnetic field will caused electromagnetic energy absorption and resulting eddy current losses, which lead to a distortion of the primary magnetic field in the vicinity of the pipeline, and formation of a weak field zone beneath the pipeline. The size and shape of the shielding zone are affected by burial depth, transmitter coil diameter, and anomaly size. When the burial depth exceeds 10 times the diameter of the coil or pipeline, the shielding range stabilizes at 1.5–2 times the pipeline’s transverse diameter. Moreover, when the pipeline’s transverse diameter exceeds twice the transmitter coil diameter, the weak field zone beneath the pipeline will transform into a strong field zone, this is due to the refractive and reflective effects of the electromagnetic field. Finally, experiments were conducted and the inverted results was found to be larger than the actual pipeline diameter, with an error margin similar to that explained by the simulation. These results have implications for high accuracy detecting underground pipelines in urban areas.

## Introduction

Urban subsurface safety has received increasing attention as urbanization has progressed more quickly [1,2]. Frequent road collapse accidents in recent years have resulted in serious injuries, property losses, and traffic jams [3]. Rapid detection technology is urgently required to look into underground diseases in urban subsurface [4,5]. Transient electromagnetic detection has the advantages of convenience and high efficiency, making it an attractive prospect for application in urban area [6,7].

Transient electromagnetic has been widely used in urban area over the last decade, particularly in small loop transient electromagnetic devices [8]. The smaller equipment volume and high efficiency make it suitable for urban underground space detection tasks. To ensure the timeliness of detection, efficient detection methods need to be combined with efficient data processing methods. The rapid cross Bayesian imaging method and pseudo-seismic wavelet transform method have shown good results [9,10]. Due to the difference in depth between urban underground space detection and field detection, different accuracies are required [11,12]. For shallow detection, data is easily affected by interference, for example, the receiver coil design has a certain correlation with the error of the apparent resistivity [13]. Therefore, it is necessary to study the interference effect in urban near-surface detection.

Geological disasters occurring in urban areas often have a sudden onset and pose a great threat, such as road collapse accidents. It is necessary to use transient electromagnetic to investigate potential hazards. The underground environment in urban areas is complex, and obtaining drilling samples is difficult due to interference effects, resulting in low accuracy in geological interpretation [14]. Therefore, numerical simulation methods are commonly used to study response patterns. Some scholars have used numerical simulation methods to investigate the response characteristics of groundwater and pile grouting [15,16], achieving good results, which indicates that numerical simulation technology has a certain degree of reliability in this field. In addition, transient electromagnetic (TEM) numerical simulation methods have advantages such as high efficiency and good visualization effects [17,18], making them suitable for studying the regularity of TEM responses.

Errors in the inversion imaging of transient electromagnetic (TEM) data are a common phenomenon, such as the errors observed when using the Gauss-Newton method to invert three-dimensional synthetic data [19]. These errors often have several characteristics: the boundaries on the left, right or lower sides of the anomalous body are larger; an interference zone appears below the anomalous body. The accuracy of transient electromagnetic inversion imaging is also closely related to the inversion algorithm. Algorithms with better fitting performance have smaller errors. For example, the MODEM inversion method has poor boundary convergence, while the Bayesian 2D inversion method performs well in boundary inversion [20,21]. However, many inversion algorithms, including machine learning algorithms, may have errors to varying degrees [22,23]. This indicates that in both practical acquisition and numerical simulation, errors are difficult to remove due to the presence of interference effects. This is a unique phenomenon of transient electromagnetic (TEM) method, as some scholars have used similar inversion algorithms to invert General Purpose Radar (GPR) data without encountering similar error phenomena [24].

Understanding the error effects in the process of transient electromagnetic (TEM) detection is essential for achieving high-precision geological investigations in urban near-surface space. This paper used finite element method to simulate the entire process of urban underground pipeline under transient electromagnetic detection. The causes of interference and the degree of interference under different working conditions were analyzed. Finally, field testing was conducted on urban roads, and data processing was performed using Bayesian inversion methods to validate the conclusions of the simulations.

## Theory

The electric field diffusion Eq (1) can be established starting from Maxwell’s equation in the time domain, assuming that the displacement current term is disregarded. Eq (1)’s weak form solution can be written as Eq (2) [25,26].


$$\frac{1}{\mu }\nabla \times \nabla \times e(r,t)+\sigma \frac{\partial e(r,t)}{\partial t}+\frac{\partial j_{s}(r,t)}{\partial t}=0$$ (1)

$${∭}_{\Omega }\left(\nabla \times v\right)\cdot (\frac{1}{\mu }\nabla \times e(r,t))d\Omega +{∭}_{\Omega }\sigma v\cdot \frac{\partial e(r,t)}{\partial t}d\Omega +{∭}_{\Omega }v\cdot \frac{\partial j_{s}(r,t)}{\partial t}d\Omega =0$$ (2)

Where *v* is the test function introduced to weight the finite element residual. *e*(*r*,*t*) is the electric field at the corresponding position and time, *j*_*s*_(*r*,*t*) is the current density, *σ* is the conductivity and *μ* is the permeability. The finite element division used the unstructured tetrahedral mesh method to discretize the diffusion equation of the electric field. The electric field of any tetrahedral element can be expressed by Eq (3) [27].


$$e(r,t)=\sum_{i=1}^{6}u_{i}^{k}(t)n_{i}^{k}(r)$$ (3)

Where $n_{i}^{k}(r)$ is the edge shape function which can automatically meet the continuity and no dispersion conditions of electric field. $u_{i}^{k}(t)$ is the tangential electric field value on the *i* edge of the *k* element, The total governing equation of finite element can be established as (4) according to Galerkin method. The boundary condition of the equation adopts the homogeneous half-space Dirichlet boundary condition. Assuming that the tangential electric field component of the outer boundary of the computational domain is 0, there is Eq (4) [28].


$$M\frac{de(t)}{dt}+Se(t)=J_{s}$$ (4)

Where *M* is the mass matrix, *S* is the stiffness matrix, *J*_*s*_ is the current source, The second order backward Euler discrete scheme is used for time discretization of Formula (4), then the unconditionally stable implicit difference equation can be obtained as Eq (5).


$$(3M+2\Delta tS)e^{m+2}(t)=M(4e^{m+1}(t)-e^{m}(t))-2\Delta tJ_{s}^{m+2}$$ (5)

The boundary condition of the equation adopts the homogeneous half-space Dirichlet boundary condition. Assuming that the tangential electric field component of the outer boundary of the computational domain is 0, it can be expressed as Eq (6), which in the case of large enough space can better reflect the actual response of the electromagnetic field.


$${\left(\hat{n}\times e\right)}_{\Gamma }=0$$ (6)

The simulated emission in this paper is a small-size loop source. The emission source can be regarded as a combination of several short wire elements, and a dense mesh is used near the source. This method adopted by Ansari and Farquharson has achieved good results and can be expressed as (7) and (8).

$$Q_{j}={∭}_{\Omega }\mu (J_{s}^{k}\cdot n_{i}^{k})dv$$ (7)

$$J_{s}^{k}(x,y,z,t)=I(t)\delta (x-x_{0})\delta (y-y_{0})\delta (z)dle_{x}$$ (8)

where $J_{s}^{k}$ is the source current density, *I*(*t*) is the time-varying current, *δ* is the lash function, *dl* is the unit length of wire source, *e*_*x*_ is the unit vector.

## Numerical calculation

### Model parameter

**Fig 1:**
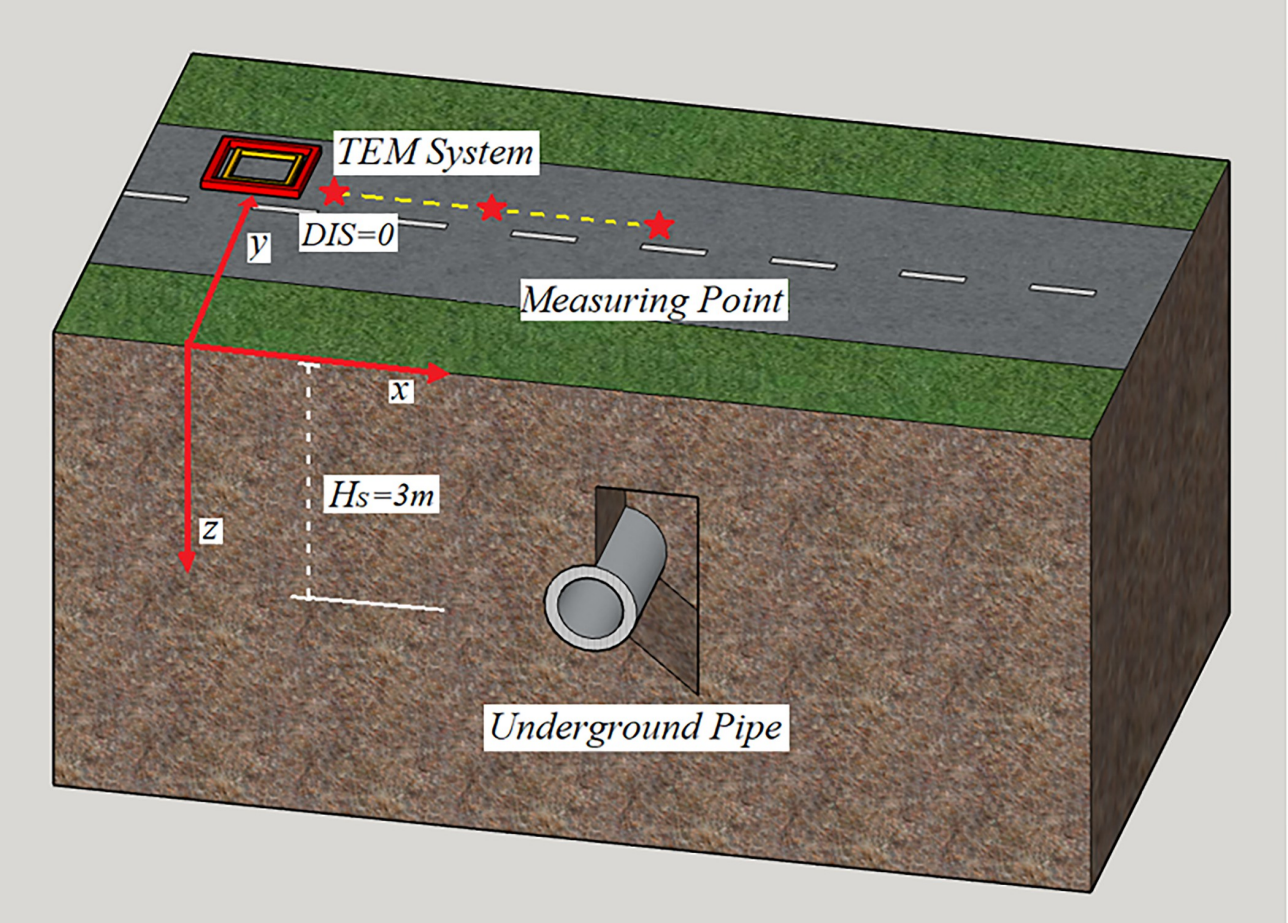
TEM pipeline response model: The TEM system’s transmitting and receiving wireframes are represented by the red and yellow wireframes, respectively, and the model-determined sampling position is the asterisk.

The transient electromagnetic response of underground pipeline depends on the resistivity of pipeline material. Metal pipeline or reinforced concrete pipeline is usually used in engineering, and the buried depth is mostly within 10m. In this study, the transient electromagnetic three-dimensional full space model of pipeline is established by software maxwell which as showing in Fig 1. There are three measurement points, with a spacing of 1m between each point. The pipeline is constructed of hollow iron with a diameter of 40cm, and the thickness of the inner wall is 10cm. The transmitting coil has a side length of 50cm and is coaxial with the receiving coil, which has a side length of 45cm. A square wave excitation with a peak value of 1A and a frequency of 25Hz is produced by the coil. The receiving and transmitting coils are located 10cm above the ground. The current lasts for 10ms, and the data acquisition continues for 10ms. The main material properties of the model are shown in Table 1.

**Table 1:** Mode materials.

Member	Materials	Resistivity(Ω·m)	Relative Permeability
Tx&Rx	copper	1.75×10^−8^	0.999991
Ground	clay	100	100
Pipe	iron	0.97×10–7	4000

### Primary field distribution characteristics

**Fig 2:**
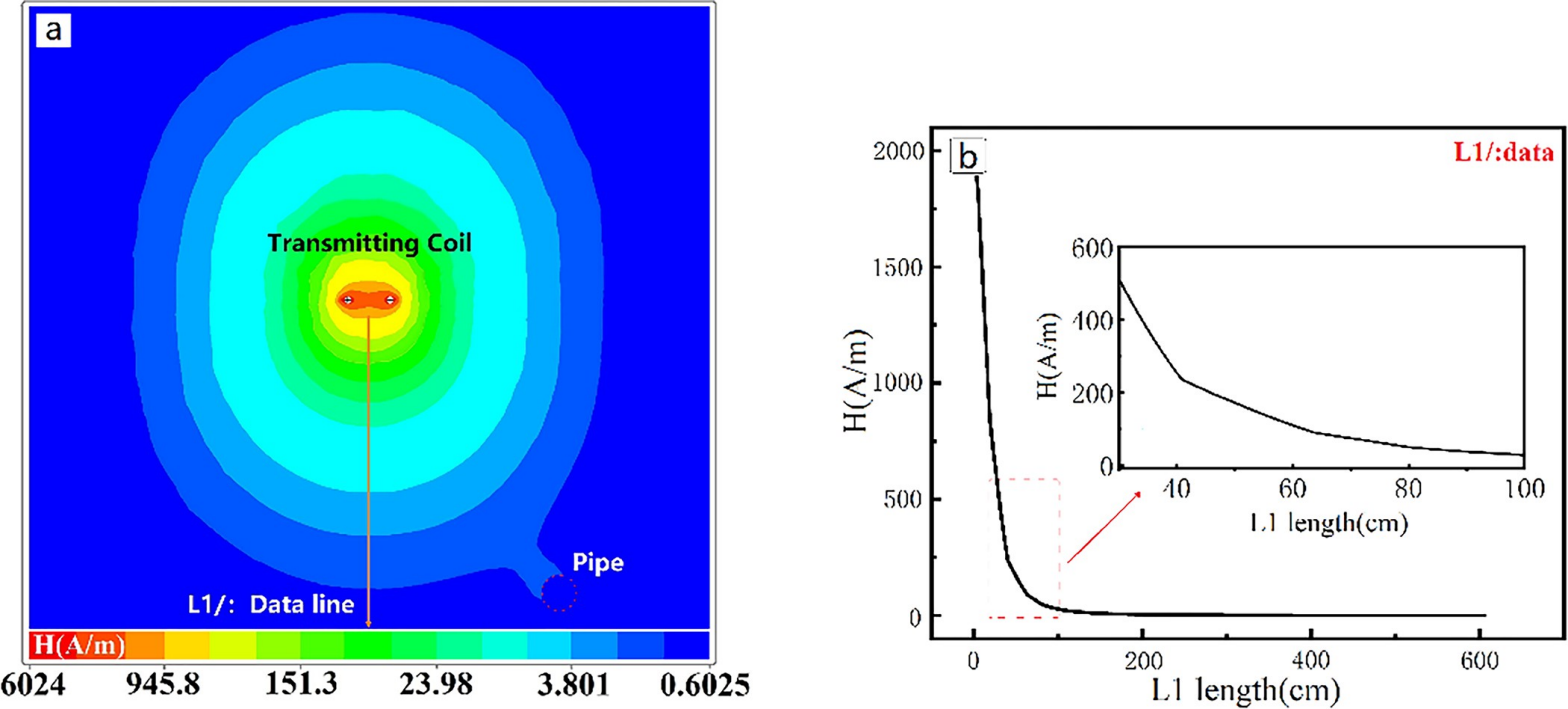
Characteristics of the TEM transmitting coil’s primary field in the presence of a pipe. a. Adhesion of the primary field to the pipe. b, primary field attenuation curve along survey line.

The TEM transmitting coil will excite a stable magnetic field when the square wave excitation current is opened. This study extracts the model’s xoy plane’s magnetic field intensity distribution, as seen in Fig 2(A). The coil is centered in the cloud map, and the magnetic field distribution is uniformly. The primary field of the square coil is mostly elliptical in the xoy plane. As can be seen from the figure, the field strength distribution of a magnetic field differs greatly. The magnetic field intensity near the transmitting coil’s center can reach 6024A/m, and the intensity of the primary field decreases exponentially with distance.

The position of the pipeline is marked in the lower right corner of the Fig 2(A). The position of the pipeline shows that the low-resistance pipeline interferes with the primary field distribution. The magnetic field distribution map of the xoy surface depicts the effect of adhesion. The low-resistance body has an adsorption effect on the magnetic field, which causes this phenomenon [29]. The influence of the low-resistance body causes some interference to the distribution of the primary field, resulting in an uneven distribution of the primary field [30]. As a result, the contour recognition and position judgment of the low-resistivity body will form interference in the subsequent data processing and inversion process.

In this paper, the magnetic field intensity in the vertical direction of the primary field is extracted for analysis (position L1/:Data line) and the results are shown in Fig 2(B). It is evident that, within 1 m of the transmitting coil, the primary field’s intensity rapidly decreases. The magnetic field intensity is 502.8A/m at 0.3m (ground position), which is 91.7% less than its peak value. The proximity of the receiving coil and the transmitting coil can result in significant interference from the primary field. This could potentially result in a lower signal-to-noise ratio in the detection of shallow subsurface features.

**Fig 3:**
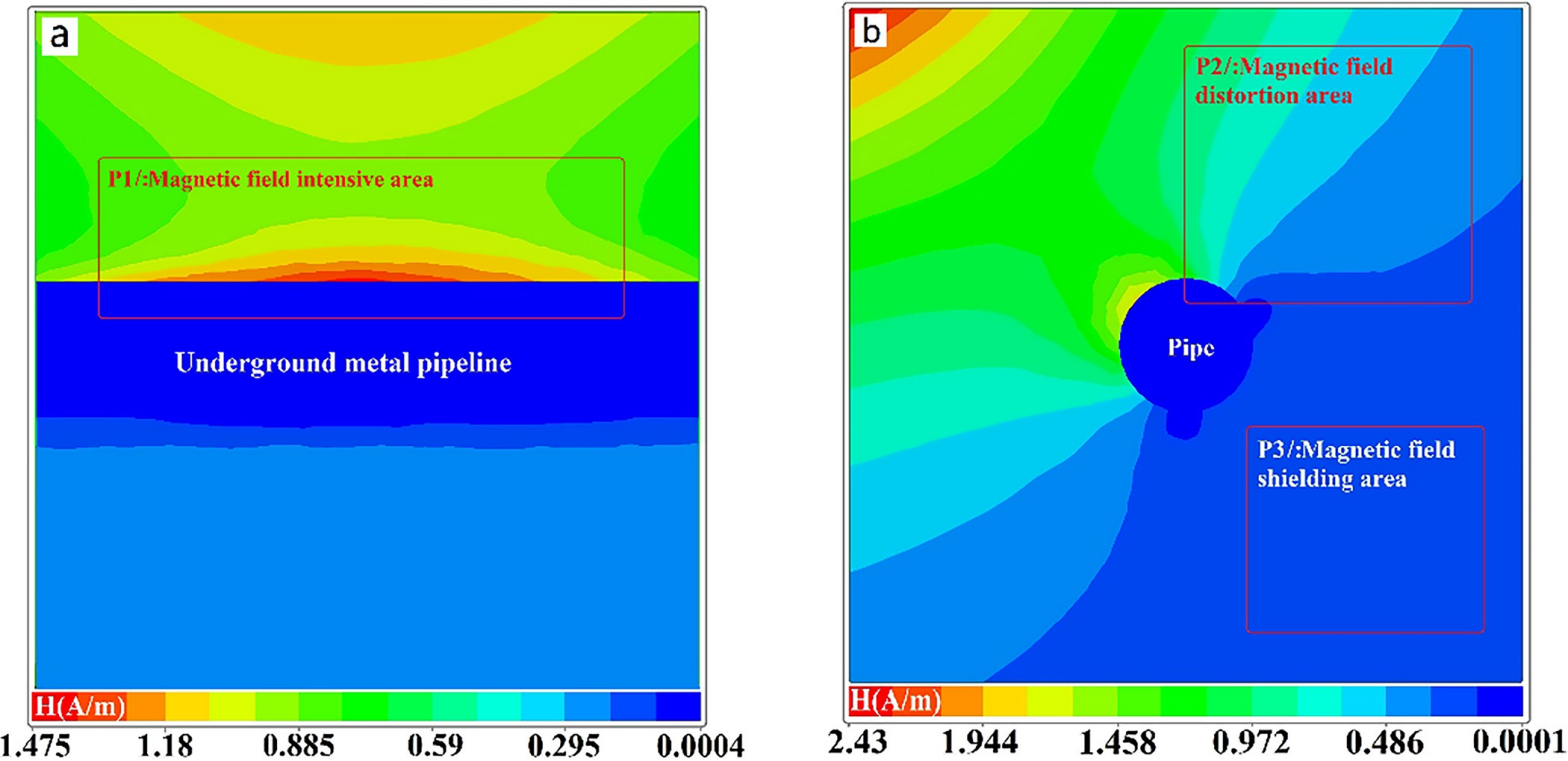
The effect of a TEM transmitting coil’s magnetic field on an underground metal pipeline. a. primary magnetic field distribution near a pipe on the xoy plane. b. primary magnetic field distribution near a pipe on the yoz plane.

The xoz plane distribution map of the primary magnetic field intensity in the pipeline section is shown in Fig 3(A). The primary magnetic field can concentrate above the pipeline, creating a region of strong magnetic field(position P1). The primary field will be shielded by the low resistance pipeline in the middle and back of pipeline. Fig 3(B) shows the distribution of magnetic field intensity in the yoz plane. The pipeline clearly distorts the distribution of magnetic field intensity, resulting in a similar effect of impeding propagation(position P2). The accuracy of detecting objects near the low resistance body may be impacted by the uneven distribution of the magnetic field that results from distortion. Under the low resistance body, the distorted magnetic field will create a low magnetic field area(position P3). The low-resistance body shields the primary magnetic field, which may affect the accuracy of the resistivity of the material being detected behind the low-resistance body. The reason for this phenomenon lies in the fact that the low resistance material has a high electrical conductivity, which absorbs electromagnetic energy from the magnetic field, leading to a loss and attenuation of magnetic field energy. This phenomenon is known as eddy current loss. This process can be explained using Ampere’s law and Faraday’s electromagnetic induction law.

### Primary field shielding effects

**Fig 4:**
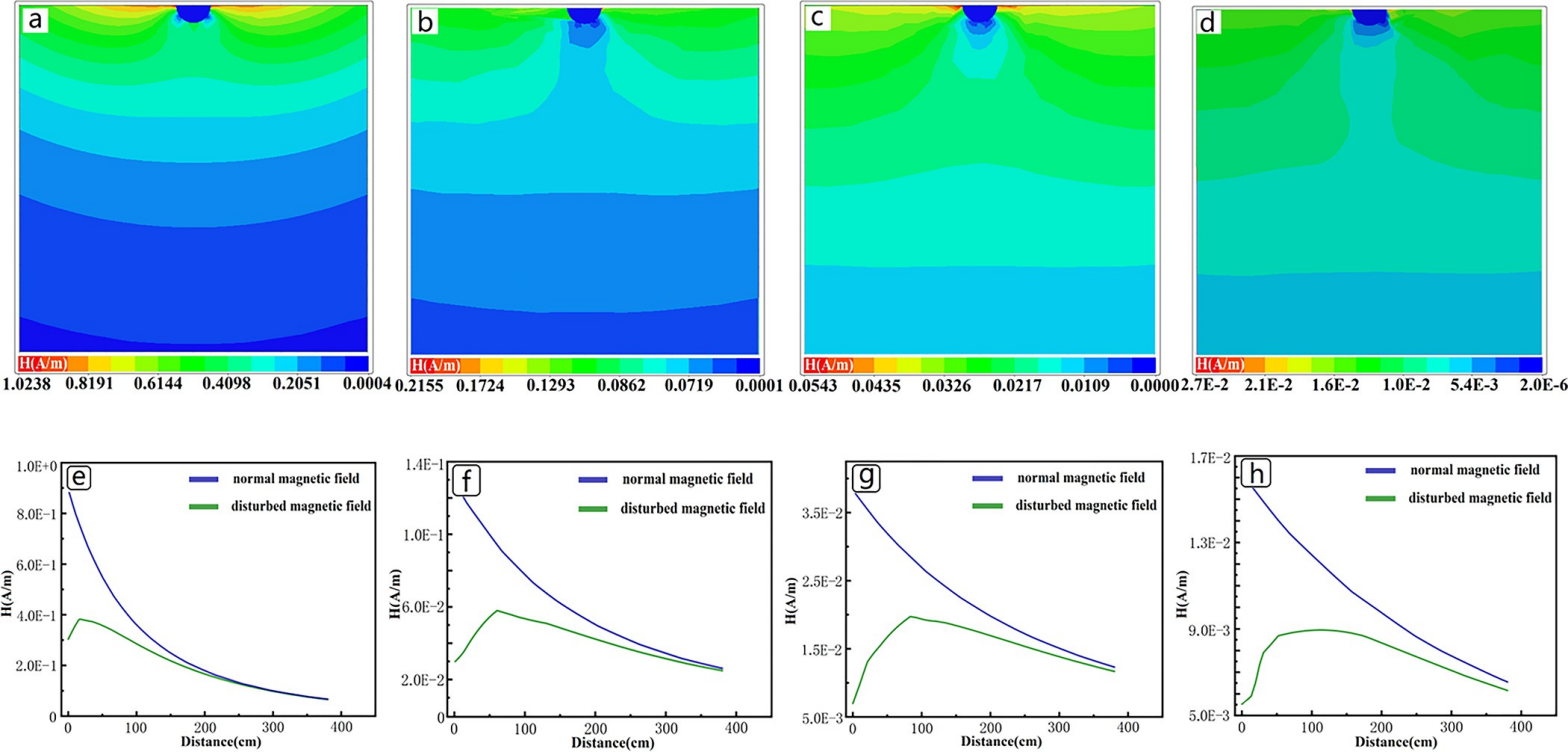
Primary field shielding range of tubular low resistance anomaly at different buried depths. (a)-(d) are the shielding conditions of the primary field when the tubular low resistance anomaly(diameter 0.4m) is buried at depths of 3m, 6m, 9m and 12m. (e)-(h) are the specific field strengths below the pipeline at different burial depths.

This paper investigates the range of the primary field shielding of pipeline under different conditions. From Fig 4(A)–4(D), it can be seen as the depth increases, the influence of the tubular low-resistance anomaly on the primary field also increases, and the affected area is mainly concentrated directly below the anomaly. When the burial depth reaches 12m, there is a trend of closure of the shielded primary field below the anomaly. This indicates that the shielding range does not continue to increase with depth; once the depth reaches a certain value, the shielding range is only related to factors such as the size of the low-resistance anomaly and the area of the transmitting coil.

The magnetic field intensity of the primary field shielding area were extracted, as shown in the resulting Fig 4(E)–4(H). At a burial depth of 3 meters, the shielding range is within 200cm directly below the anomaly, and the shielding level reaches around 77% at the position closest to the pipeline. With the increase of depth, the shielding range is increasing, but the maximum shielding degree is not changing much, maintaining between 65–85%. When the depth reaches 12m, the influence depth exceeds the lateral diameter of the pipeline, and the final influence depth is expected to be 1.5–2 times the lateral diameter of the pipeline. As the depth increases, the shielding range is also increasing, the maximum shielding degree change is not large. When the depth reaches 12m, the interference depth exceeds the transverse diameter of the low-resistance anomalous body, the final interference depth is expected to be 1.5–2 times the transverse diameter of the anomalous body.

**Fig 5:**
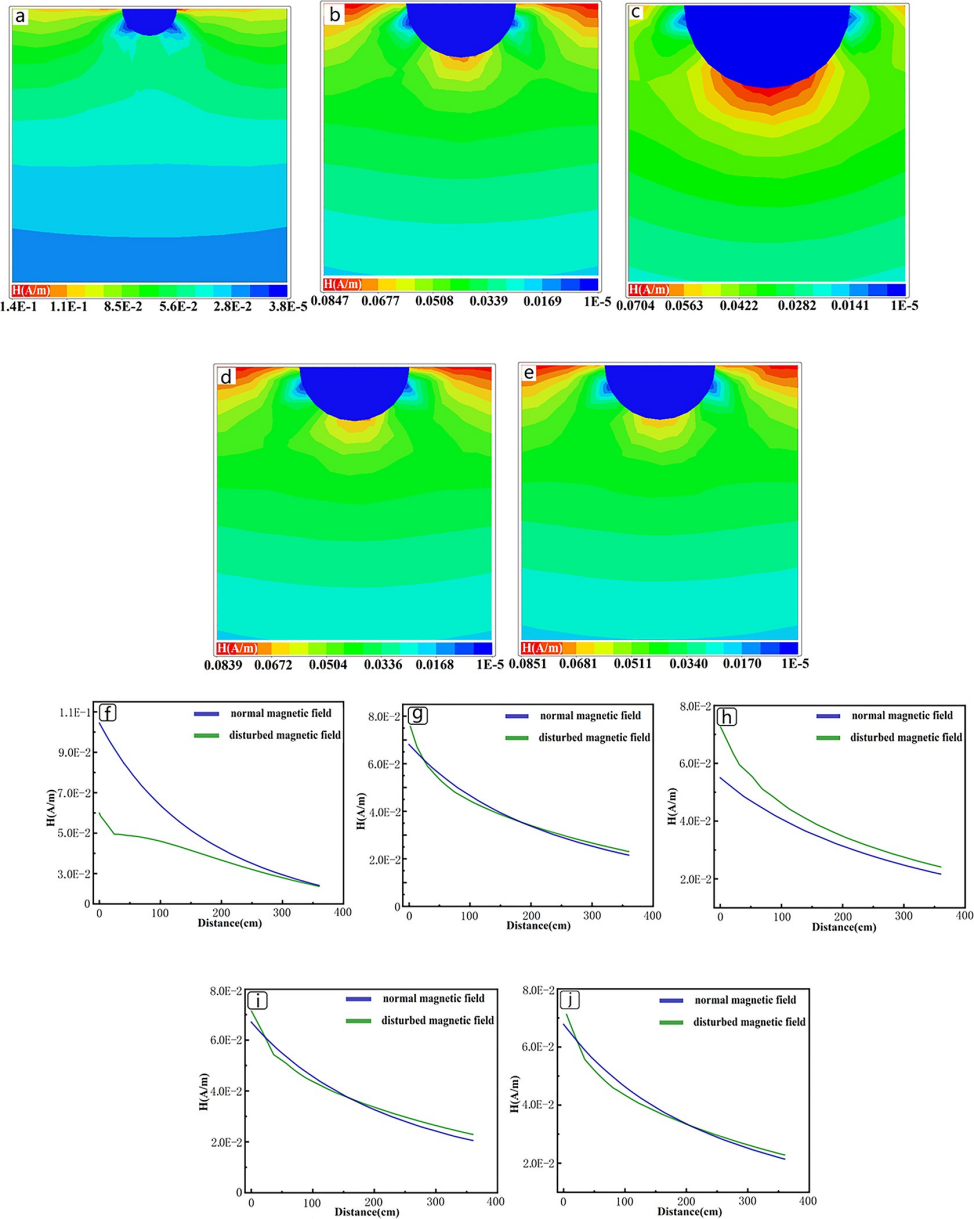
Primary field shielding range of tubular low resistance anomaly with different thickness and radius. The diameters of (a)-(c) are 0.8m, 1.6m, and 3.2m, respectively, with a thickness of 0.1m and a burial depth of 6m. The diameter of (d)-(e) is 1.6m, the thickness is 0.2m, 0.4m, and the burial depth is 6m. (f)-(j) are the specific field strengths below the corresponding pipeline.

The correlation between the buried depth and the primary field shielding effect will decrease as the buried depth increases, and the greater correlation should be the parameter of the low resistance body itself. Fig 5(A)–5(C) shows the primary field response of pipeline with different diameters(thickness not change). When the diameter changes from 0.8m to 1.6 m, the strong shielding area changes from directly below the pipeline to the lower sides. As the diameter increases, a strong magnetic field region appears directly below the pipe, which indicates that the mechanism of shielding will change when the diameter of the low-resistance anomaly is larger than the diameter of the emission coil. As can be seen from Fig 5(F)–5(H), with the increase of the diameter, the magnetic field below the pipeline will also become stronger, and the ratio of the transverse length of the anomaly to the diameter of the emission coil will lead to the occurrence of this phenomenon. The cause of this effect is due to the difference in electromagnetic properties between two media as electromagnetic waves propagate through them. The propagation velocity of the magnetic field is different in these media, resulting in the reflection and refraction of the electromagnetic field in the presence of low resistance materials. This creates a strong field region beneath the low resistance material. This may lead to the accompanying anomaly of high resistance under the large low resistor in the inversion calculation.

Fig 5(D), 5(E), 5(I) and 5(J) show the primary magnetic field response of pipeline with different thicknesses, and it can be seen that the gap between their response results is small, which indicates that for tubular low resistance anomalies, thickness has little effect on the shielding effect.

### Secondary field response

**Fig 6:**
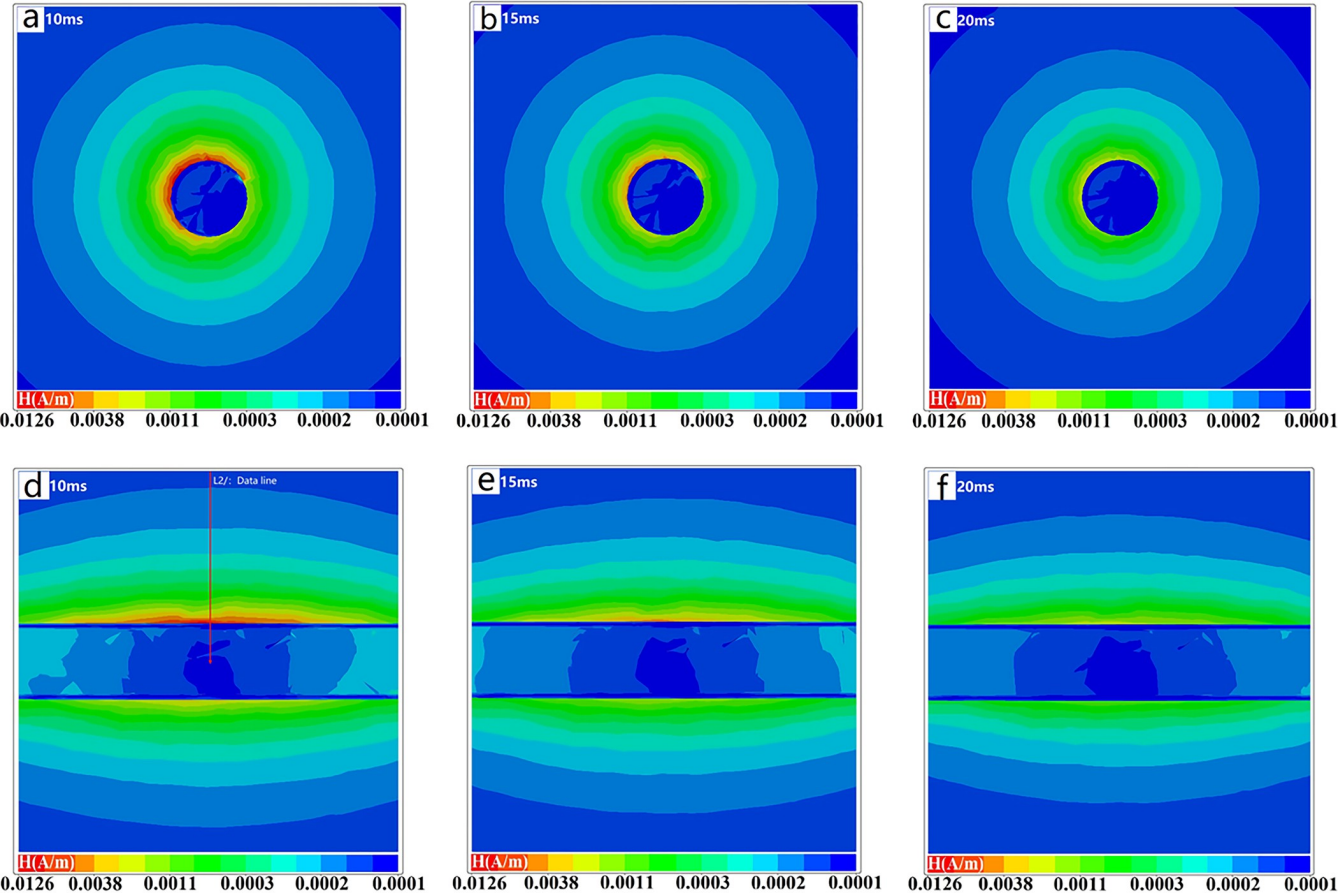
Distribution of secondary magnetic field near pipe in yoz Plant. a. Intensity distribution of secondary field at 10 ms, in yoz Plant b. Intensity distribution of secondary field at 15 ms, in yoz Plant c. Intensity distribution of secondary field at 20 ms, in yoz Plant d. Intensity distribution of secondary field at 10 ms, in xoy Plant e. Intensity distribution of secondary field at 15 ms, in xoy Plant f. Intensity distribution of secondary field at 20 ms, in xoy Plant.

The secondary field is more evenly distributed than the primary field, as shown in Fig 6(A)–6(C). The secondary field will have some areas of high magnetic field intensity near the transmitting coil position. It can be seen that the distribution of the secondary magnetic field generated after the square wave excitation of the transmitting coil is not uniform, which may be related to the material properties of the inductive effect products, and the uneven secondary field may affect the received data results. As time increases, the high magnetic field intensity area attenuates, and finally the secondary magnetic field intensity distribution will return to uniform. The distribution of the secondary magnetic field excited by different coil positions differs, and the distribution of the magnetic field contains shape and position information. This may require us to use a more anti-interference receiving method and a more accurate inversion method to receive and analyze.

Fig 6(D)–6(F) depicts the secondary magnetic field distribution in the xoz plane. A certain correlation is presented between peak value around the pipeline edge and the position of the transmitting coils. The closer to the coil, the greater intensity of the magnetic field. Thus, when data acquisition is conducted, it is difficult to precisely distinguish the contours of anomalies in 3D or 2D transversal inversion process if the unevenness of this part of magnetic field distribution is not included in the TEM inversion calculation. The interior of the pipeline is high-resistance air, and the distribution of magnetic field intensity inside the pipeline is quite different from that outside the pipeline due to the shielding effect of the low-resistance pipeline. This demonstrates that, under the influence of the low-resistance pipeline’s shielding effect, the abnormal body in the pipeline is difficult to identify. The magnetic field intensity distribution inside the pipeline has no discernible attenuation trace as time passes, which may be due to the induction eddy current of the surrounding low resistance pipeline delaying the attenuation of the internal magnetic field.

**Fig 7:**
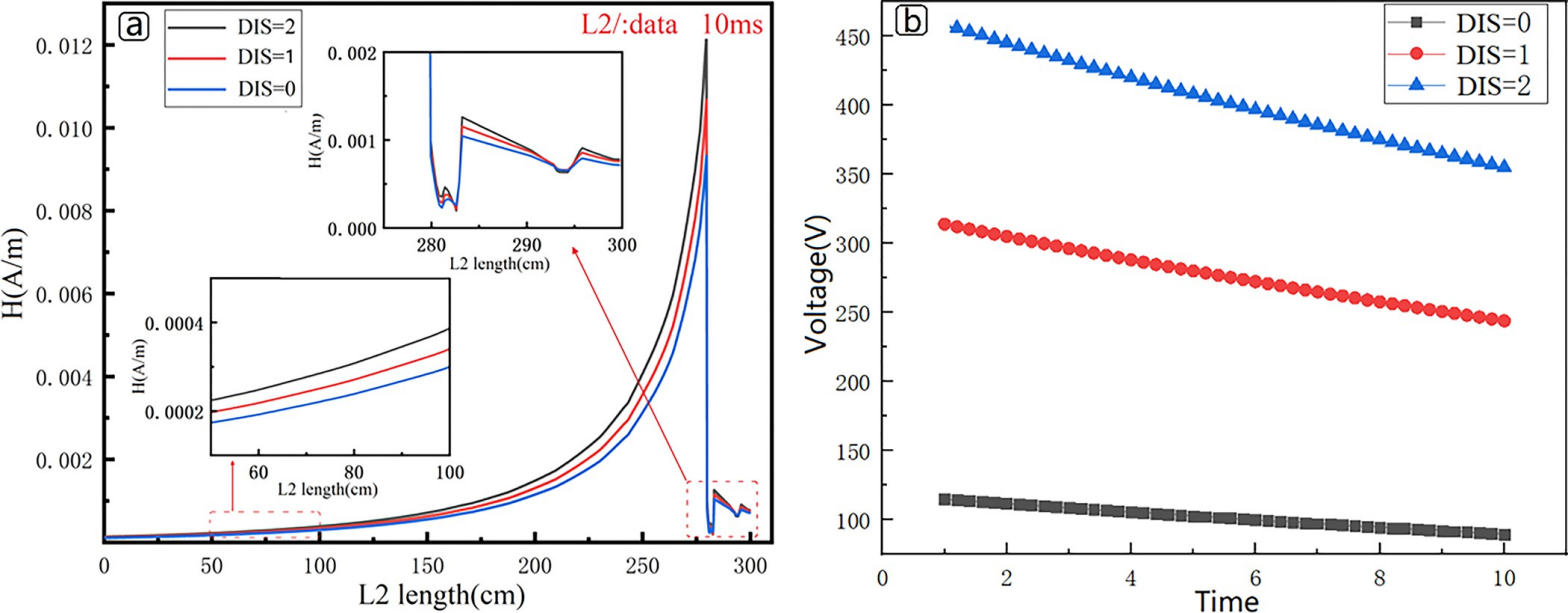
Numerical simulation response results. a. The magnetic field intensity which extracted in Fig 6D (position L2/:Data line). b. Time-voltage curves received by the receiving coil at different sampling positions.

To better understand the characteristics of the secondary magnetic field of the underground low-resistance anomaly at various times during the receiving process, the magnetic field intensity in Fig 6D is extracted for analysis (position L2/:Data line) and the results are shown in Fig 7(A) The intensity of the secondary magnetic field increases exponentially from the ground to the pipeline’s outer surface. Because of the high conductivity of the material, the intensity of the secondary field decreases cliff-like when it reaches the pipeline body. Due to the shielding effect of the pipeline, the magnetic field presents an irregular zigzag when it reaches the inside of the pipeline, and the closer to the center of the pipeline, the lower the magnetic field intensity. The magnitude of the secondary magnetic field of the pipeline varies slightly at different positions. The largest response is observed directly above the pipeline, and the response decreases by approximately 20% at a position offset by 2 meters. Near anomalous bodies, side effects can have a significant impact.

Following that, as shown in Fig 7(B), this paper analyzes the time-voltage signals collected at various locations. The closer you are to the pipeline, the higher the collected voltage value, and data collected at sampling point DIS = 2 is on average 45% higher than data collected at DIS = 1, and data collected at DIS = 1 is on average 172% higher than data collected at DIS = 0. Aside from the average voltage value, the sampling points are different, as is the slope of the data. The slope of the time voltage curve increases as one gets closer to the low resistance anomaly point.

## Cases study

**Fig 8:**
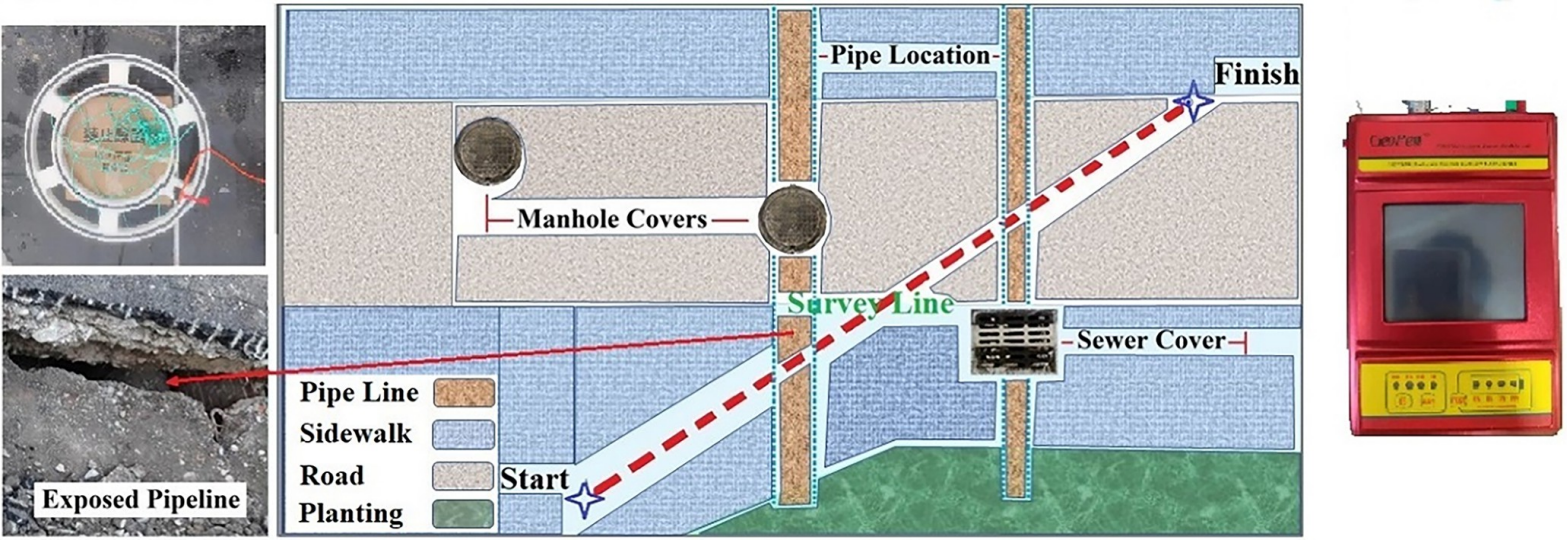
The figure details the specific experiment environment, with two pipes along the detection path, the manhole covers with potential interference marked on site. The TEM coil is in the top left of the figure and the actual photo of the pipe is in the bottom left. Device pictures are shown on the right side.

This paper presents experiments on urban roads, the experiments content is to use TEM small loop device to detect the pipeline, analyze the measured data and the inversion results. The GEOPEN company’s TEM31 apparatus was utilized. The sampling frequency was 25Hz, and the emission current was 1A. The transmitting coil’s side length was 50 cm, and the receiving coil had a side length of 45 cm. The road in Chaoyang District, Changchun City, Jilin Province, China, was chosen as the test site. The underground pipeline situation in the area has been clearly understood. At a depth of roughly 1m below the road, there was a metal bellows with a diameter of about 30 cm and a rainwater pipe with a diameter of about 60 cm. Equidistant sampling was used to sample the equipment every 30 cm. The sampling route measured 16.8 m in length and had a total of 56 data points. During the detection process, efforts are made to minimize the noise errors caused by human movements and vehicles. Fig 8 depicts the exact test route and field environment. The position of the pipeline route to be measured in the figure is annotated, as well as that of the road, greenbelt and pedestrian crossing. Since the material of the manhole cover is cast iron, it will cause interference to the transient electromagnetic device. The measurement route of this measurement avoided the position of the manhole cover as much as possible, and the distance between the measuring equipment and the manhole cover was more than 3m.

**Fig 9:**
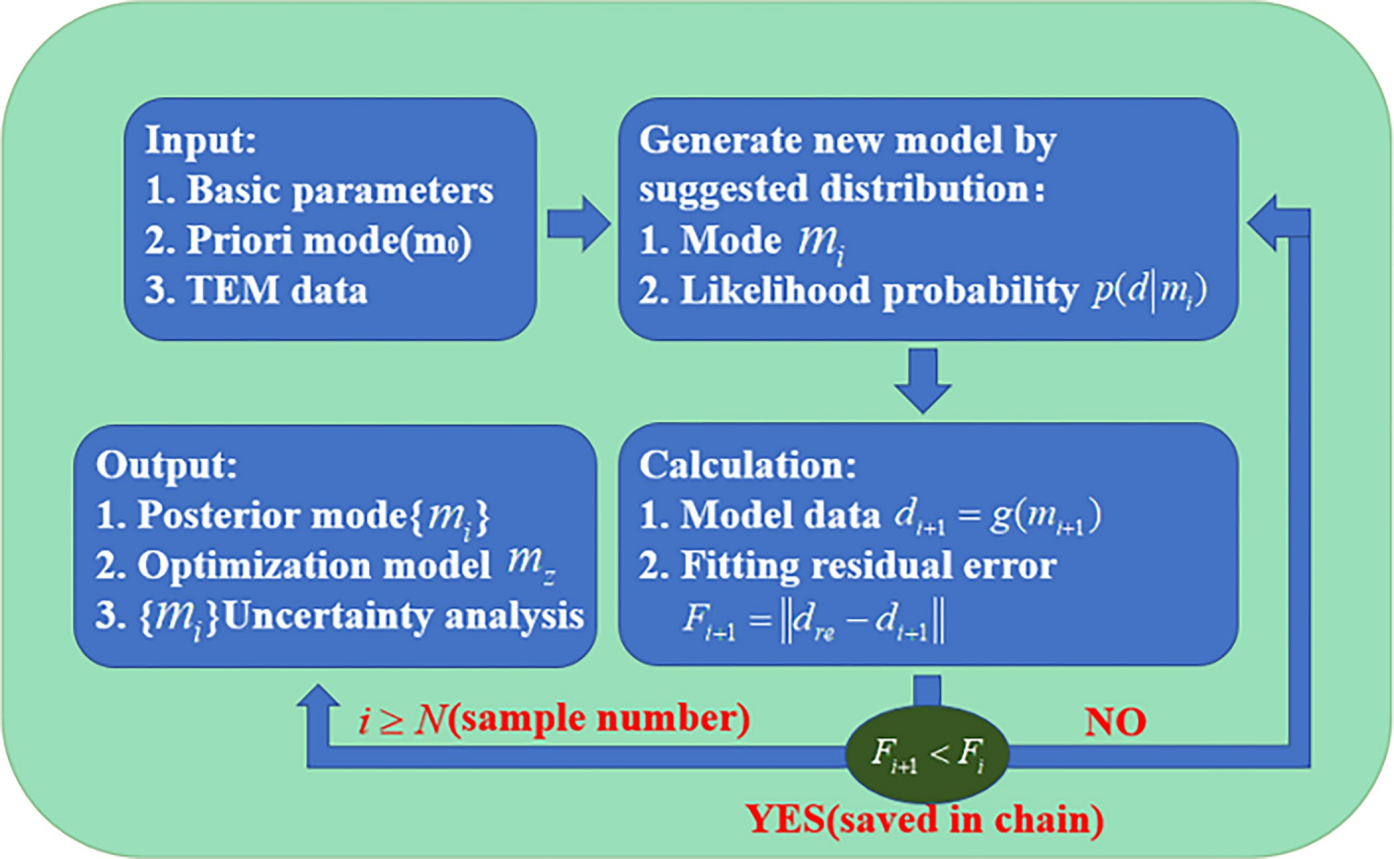
Bayesian inversion method diagram: Contains input, generate model, calculation, output parts.

The same measuring point will be taken 8 times to ensure data accuracy, and then the collected data will be superimposed to remove unintentional errors. This paper adopts the quasi-2-dimensional Bayesian method to invert the transient electromagnetic data. The Bayesian inversion method can statistically provide the best models of resistivity interpretation and avoid wrong inversion results due to multi-solution problems. Scholars have used the Bayesian inversion method to process TEM data, and the inversion effects are satisfactory [31–33]. The inversion flow of the Bayesian method is shown in Fig 9.

**Fig 10:**
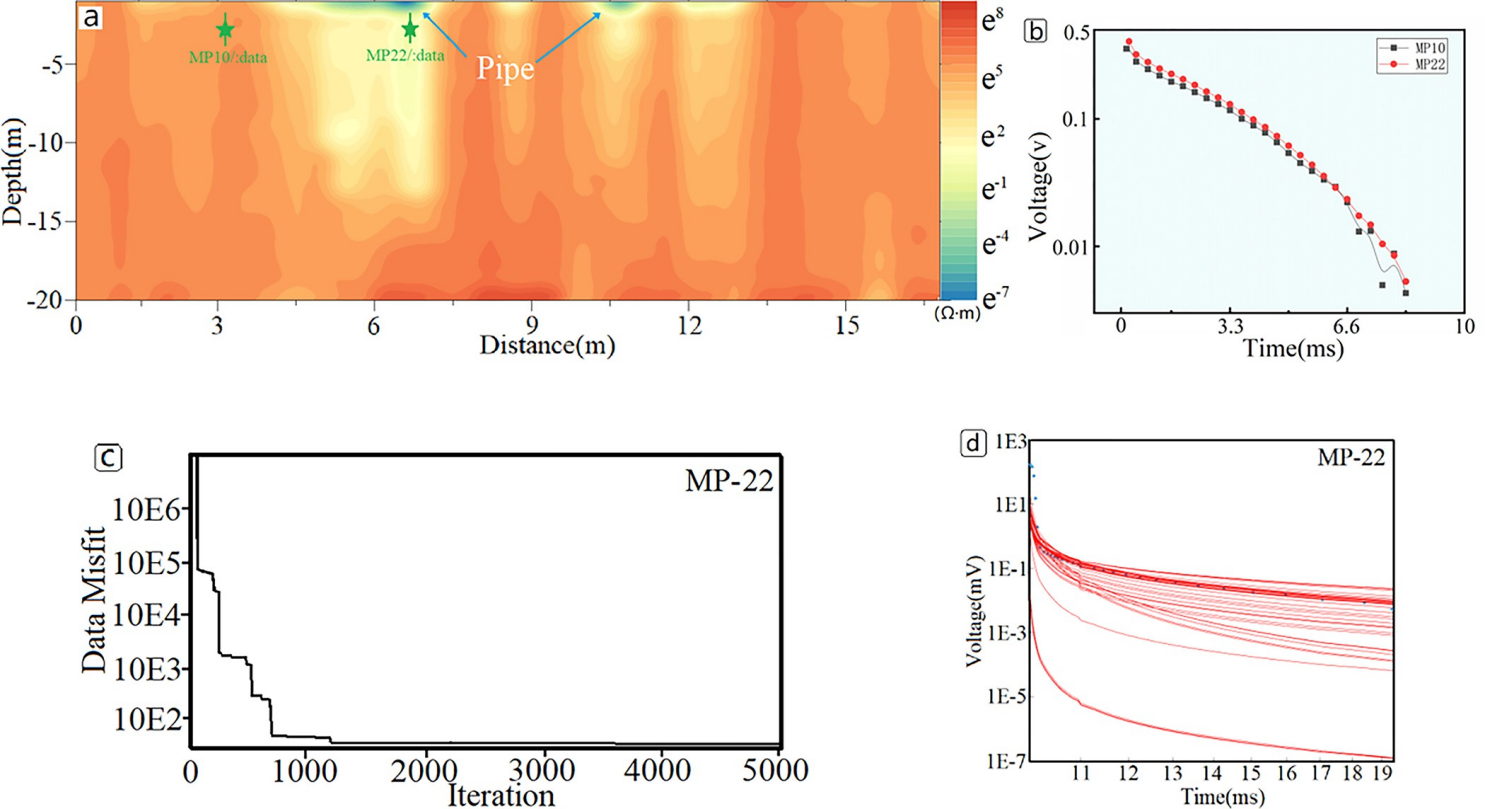
Inversion results and field data. (a)The resistivity profile after Bayesian inversion: The figure indicates the locations of known pipes, areas of anomalous resistivity, and locations of measured data from related studies. (b)Upon comparison of the experimental data from locations PM10 and PM22 in Fig 10(A), no pipeline information was found in location PM10, whereas pipeline information was present at location PM22. (c)The iteration misfit of the MP-22 Bayesian inversion for the data was computed using 5000 samples. (d) The fitting performance of the data MP-22 Bayesian inversion.

Results from Bayesian inversion are shown in Fig 10(A), where the locations of pipes in response are basically consistent with those of pipes in the field experiment map. From the figure, the pipeline does not show a regular circular shape, and the boundaries on both sides are significantly larger than actual, which is due to the interference effect of lateral measurement. The larger the transverse diameter of the low-resistance body, the stronger the lateral interference effect. After inversion, the lower boundary of the pipeline is also larger than actual and approximately equal to twice the actual value, which is consistent with the simulated results. A low-resistance area appears below the pipeline, which may be influenced by the fitting effect of the inversion algorithm or the lower resistivity of the soil caused by water content.

This study compares the response results with and without pipelines after extracting two original data sets (MP10 and MP22 data). The voltage response value will be higher when there are pipelines, as shown in Fig 10(B), and the slope of the data at the beginning of the curve will also be higher, which is consistent with the simulation results presented above. Because the measured results represent a mixed response of the primary field and the secondary field, whereas the simulation represents only the response of the secondary field, the difference between the simulation results and the measured results is greater. Fig 10(C) shows the iteration error of the MP-22 data samples. It can be observed that as the number of samples exceeds 1000, the iteration error significantly decreases, indicating a better inversion performance. The fitting between the inversion data and the measured data is illustrated in Fig 10(D). It can be observed that, apart from the first three data points, the overall fitting of the data is satisfactory. The reason for the larger values of the first three data points is attributed to significant interference from the primary field.

## Discussion

Urban geological disasters are sudden and dangerous, necessitating the use of remote sensing technology for disease detection [1,2]. The high efficiency of transient electromagnetic detection can meet the detection requirements [7], but there is no mature TEM road disease detection system, so additional research is required. The investigation of geological hazards in urban near-surface space requires high accuracy. Therefore, we used the finite element method to analyze the characteristics and range of errors and validated them through experiments. This provides some reference and theoretical support for urban shallow geological interpretation. Subsequent research should focus on improving the efficiency and accuracy of detection, eliminating errors, forming a mature system, and widely applying it to engineering practice.

## Conclusion

This paper employs the finite element numerical simulation method to study the characteristics and range of errors generated during the TEM urban shallow detection process. The results are verified through field experiments. The following conclusions are drawn:

1. Due to the low-conductive anomaly in magnetic field will caused electromagnetic energy absorption and resulting eddy current losses, the underground pipelines can significantly distort the distribution of the magnetic field intensity, resulting in a similar obstructive propagation effect. Under the influence of shielding effect, the anomalies in the pipeline are difficult to identify.
2. The size and shape of the shielding area are influenced by burial depth, transmitter coil diameter, and anomaly size. When the burial depth exceeds 10 times the diameter of the coil or pipeline, the shielding range stabilizes at 1.5–2 times the transverse diameter of the pipeline. When the transverse diameter of the pipeline exceeds twice the diameter of the transmitter coil, the weak field area below the pipeline will transform into a strong field area, this is due to the refractive and reflective effects of the electromagnetic field.

## Supporting information

S1 Data Data of underground pipelines detected using transient electromagnetic methods (https://figshare.com/articles/dataset/data_xls/23306234). (XLS)
